# Exercise suppresses tumor growth through epinephrine- and IL-6-dependent mobilization and redistribution of NK cells

**DOI:** 10.1186/2051-1426-3-S2-P246

**Published:** 2015-11-04

**Authors:** Line Pedersen, Manja Idorn, Gitte Holmen Olofsson, Intawat Nookaew, Rasmus Hvass Hansen, Helle Hjorth Johannesen, Jürgen C Becker, Britt Lauenborg, Katrine S Pedersen, Christine Dethlefsen, Jens B Nielsen, Julie Gehl, Bente Klarlund Pedersen, Per thor Straten, Pernille Hojman

**Affiliations:** 1 Centre of Inflammation and Metabolism and Centre of Physical Activity Research, Rigshospitalet, Faculty of Health Science, University of Copehagen, Copenhagen, Denmark; 2 Centre for Cancer Immune Therapy, Dept. of Hematology, Copenhagen University Hospital, Herlev, Denmark; 3 Dept. of Chemical and Biological Engineering, Chalmers University of Technology, Göteborg, Sweden and Comparative Genomics Group, Biosciences Division, Oak Ridge National Laboratory, Oak Ridge, Tennessee, 37831, USA, Göteborg, Sweden; 4 Dept. of Radiology, University Hospital Copenhagen, Herlev, Denmark; 5 Dept. for Translational Skin Cancer Research (TSCR) within the German Cancer Consortium (DKTK), Westdeutsches Tumorzentrum, University Hospital Essen, Essen, Germany; 6 Dept. of Chemical and Biological Engineering, Chalmers University of Technology, Göteborg, Sweden; 7 Dept. of Oncology, Copenhagen University Hospital, Herlev, Denmark, Herlev, Denmark; 8 Centre for Cancer Immune Therapy (CCIT), Copenhagen University Hospital Herlev, Herlev, Denmark

## 

Regular exercise reduces the risk of cancer and disease recurrence. Yet the mechanisms behind this protection remain to be elucidated. In this study, tumor-bearing mice randomized to voluntary wheel running showed significant exercise related reduction in tumor incidence and growth across several tumor models including transplantable tumors (Lewis lung and B16 melanoma), chemically (diethylnitrosamine (DEN) induced liver cancer, and a model of spontaneous melanoma (Tg(Grm1)EPv transgenic mice). Microarray analysis revealed exercise-induced up-regulation of pathways associated with immune function, prompting further investigations. NK cell infiltration was significantly increased in tumors from exercising mice, and depletion of NK cells by anti-asialo-GM1 administration increased tumor growth and blunted the exercise-dependent tumor suppression. Mechanistic analyses showed that NK cells were engaged through an epinephrine-dependent mobilization, and blockade of this response by ß-adrenergic blockade blunted the exercise-dependent tumor inhibition. Moreover, exercise-induced IL-6 facilitated redistribution of NK cells to peripheral tissues and induced a shift towards more cytotoxic (CD11b-, CD27+) NK cells at the tumor site. Together these results link exercise, epinephrine and IL-6 to NK cell mobilization and activation, and ultimately to improved control of tumor growth.

